# Supplementary material for: Microbial Communities of Artisanal Fermented Milk Products from Russia
Source: Microorganisms. 2022 Oct 29;10(11):2140. doi: 10.3390/microorganisms10112140 (PMC9697859; doi:10.3390/microorganisms10112140)
Supplement: Supplementary file 1 [file microorganisms-10-02140-s001.zip › microorganisms-1954227-supplementary.pdf]

SUPPLEMENTARY MATERIAL

Supplementary Material Content

Supplementary Table S1. Accession numbers of SRA used in this work (excel file).

Supplementary Table S2. Concentration of volatile fatty acids (mM) in fermented milk products.

Supplementary Table S3. Resulting number of reads per sample after filtering, denoising and chimera removal (excel file).

Supplementary Figure S1. Rarefaction curves calculated for 55 fermented milk product samples. A - curves for aarts (AA), khurunga (KR), koumiss (KM) and shubat (SB) samples; B – curves for ayran (AR) and kefir (KF) samples; C – curves for prostokvasha (PS) samples; D – curves for matsoni-like product (MC), ryazhenka (RZ), tan (TA) and chegen (CG) samples; E – curves for sour-cream (SN+SM) samples; F – curves for cottage cheese (TG) samples; G – curves for bryndza (BZ) samples; H – curves for suluguni-like cheese (SU) and khuruud (KU) samples.

Supplementary Figure S2: FMP microbiomes dissimilarity displayed by NMDS plots (stress value = 0.13). Color grading corresponds to the concentrations of lactate (A), acetate (B), succinate (C), propionate (D), formate (E), *n*-butyrate (F). Gray color of samples indicates that VFA concentration was not measured in these samples. Red vectors indicate the correlation of the dominant genera abundance with the axes of ordination and their statistical significance based on a permutation test (1000 permutations, P-value < 0.05) i. e., demonstrate the strength and direction (within the present coordinates) of influence of certain bacterial taxa on samples clustering.

Supplementary Figure S3. Distribution of the 62 most abundant ASV on the genus level for beverages.

Supplementary Figure S4. Distribution of the 62 most abundant ASV on the genus level for cheeses and creamy products.

Supplementary Table S2. Concentration of volatile fatty acids (mM) in fermented milk products.

Sample type	Location	Sample acronym	Succinate	Lactate	Formate	Acetate	Propionate	n-Butyrate	Total VFA
			mM						
**Beverages**									
Aarts (AA)	Buryatia Republic	25AA	6.79	293.26	1.23	64.28	0.00	0.00	365.55
Khurunga (KR)	Buryatia Republic	18KR	0.00	188.31	0.00	58.33	0.00	0.00	246.64
		26KR	4.83	233.64	0.72	43.26	0.00	1.41	283.86
Koumiss (KM)	Buryatia Republic	31KM	10.86	206.03	0.00	45.83	0.00	0.00	262.71
Ayran (AR)	Karachay-Cherkessia Republic	3AR	0.42	149.20	0.00	13.07	0.21	0.00	162.90
		5AR	0.20	174.82	0.00	9.45	0.00	0.00	184.47
	Stavropol Region	6AR	0.21	88.42	0.00	12.27	0.42	0.00	101.31
		13AR	1.16	102.39	0.47	7.68	0.23	0.00	111.94
		15AR	0.00	97.30	0.00	19.46	0.78	0.00	117.54
	Altai Republic	78AR	*	*	*	*	*	*	*
Kefir (KF)	Stavropol Region	7KF	0.00	167.14	0.00	8.21	0.20	0.40	175.95
	Primorsky Region	25KF	0.98	86.46	0.59	26.33	0.20	0.00	114.56
Prostokvasha (PS)	Arkhangelsk Region	16PS	0.59	176.06	1.96	22.40	0.20	0.00	201.21
	Tula Region	17PS	0.00	162.81	2.23	27.26	0.00	0.00	192.30
	Dagestan Republic	29PS	12.82	126.35	0.94	68.18	5.94	0.00	214.23
		37PS	8.56	82.36	1.60	36.90	11.23	0.00	140.65
		46PS	6.46	116.68	0.97	56.24	1.29	0.00	181.65
		66PS	5.28	190.73	1.13	29.40	2.26	0.00	228.80
		69PS	4.76	165.86	1.10	30.02	0.00	0.00	201.74
		74PS	0.00	123.08	1.05	0.00	2.80	0.00	126.93
Ryazhenka (RZ)	Stavropol Region	13RZ	0.63	451.54	0.32	31.69	0.00	0.00	484.18
	Tula Region	17RZ	0.00	249.62	0.00	19.09	0.55	0.00	269.26

	Primorsky Region	25RZ	0.00	213.45	0.00	216.47	3.22	0.00	433.14
Shubat (SB)	Altai Republic	77SB	*	*	*	*	*	*	*
Tan (TA)	Primorsky Region	22TA	0.00	18.95	1.80	4.99	0.60	0.00	26.33
**Creamy products**									
Chegen (CG)	Altai Republic	75CG	*	*	*	*	*	*	*
		76CG	*	*	*	*	*	*	*
Matsoni-like (MC)	Karachay-Cherkessia Republic	5MC	0.29	93.78	0.00	9.18	0.57	0.00	103.82
	Stavropol Region	13MC	1.66	126.69	0.55	9.70	0.28	0.28	139.16
Sour cream (SN+SM)	Stavropol Region	6SN	2.08	192.13	0.00	53.41	5.55	0.00	253.17
		7SN	1.40	127.76	0.00	50.54	6.32	0.00	186.02
		15SN	11.26	159.71	0.00	45.03	1.41	0.00	217.41
	Kabardino-Balkarian Republic	14SN	1.32	149.46	0.00	55.55	5.29	0.66	212.28
	Tula Region	20SN	6.22	296.10	0.00	77.14	7.46	0.00	386.92
	Buryatia Republic	20SM	8.80	134.02	2.97	18.46	5.21	0.00	169.46
		22SM	8.88	249.58	4.35	75.41	0.00	0.00	338.21
Cottage cheese (TG)	Karachay-Cherkessia Republic	3TG	0.56	224.72	0.00	153.17	3.91	0.00	382.36
		5TG	5.00	317.46	0.00	56.24	0.62	0.00	379.33
	Stavropol Region	6TG	0.44	248.14	0.00	29.60	2.61	0.00	280.79
		7TG	8.01	399.62	0.00	71.38	1.33	0.00	480.34
		15TG	0.00	323.91	0.60	79.16	17.52	0.00	421.20
	Kabardino-Balkarian Republic	14TG	4.48	305.93	0.00	96.26	0.75	0.00	407.41
	Arkhangelsk Region	16TG	0.49	147.87	0.00	13.35	0.00	0.49	162.21
	Tula Region	17TG	2.59	68.99	0.00	18.11	1.29	0.00	90.98
		20TG	5.91	215.65	0.00	41.36	1.18	0.00	264.10

	Buryatia Republic	30TG	47.94	238.69	3.05	60.55	0.00	0.00	350.23
**Cheeses**									
Bryndza (BZ)	Karachay-Cherkessia Republic	3BZ	0.00	813.64	3.66	76.24	5.49	0.00	899.03
		5BZ	0.00	662.71	0.00	69.97	3.04	0.00	735.72
	Stavropol Region	7BZ	0.49	350.96	0.00	65.47	0.98	0.00	417.90
		15BZ	0.00	539.41	0.59	74.97	0.59	0.00	615.55
	Kabardino-Balkarian Republic	14BZ	0.00	626.80	0.55	83.10	0.00	0.00	710.45
Suluguni-like salt cheese (SU)	Karachay-Cherkessia Republic	3SU	1.77	448.35	0.59	69.52	0.00	0.00	520.22
		5SU	3.34	213.75	0.00	74.15	0.00	0.00	291.24
	Stavropol Region	6SU	38.79	368.97	0.00	122.41	3.45	0.00	533.62
Khuruud (KU)	Buryatia Republic	29SR	5.67	126.41	0.00	31.81	0.00	0.00	163.89

*- VFAs concentration of samples from Altai Republic (Ayran 78AR, Chegen 75CG, 76CG and Shubat 77SB) was not measured.

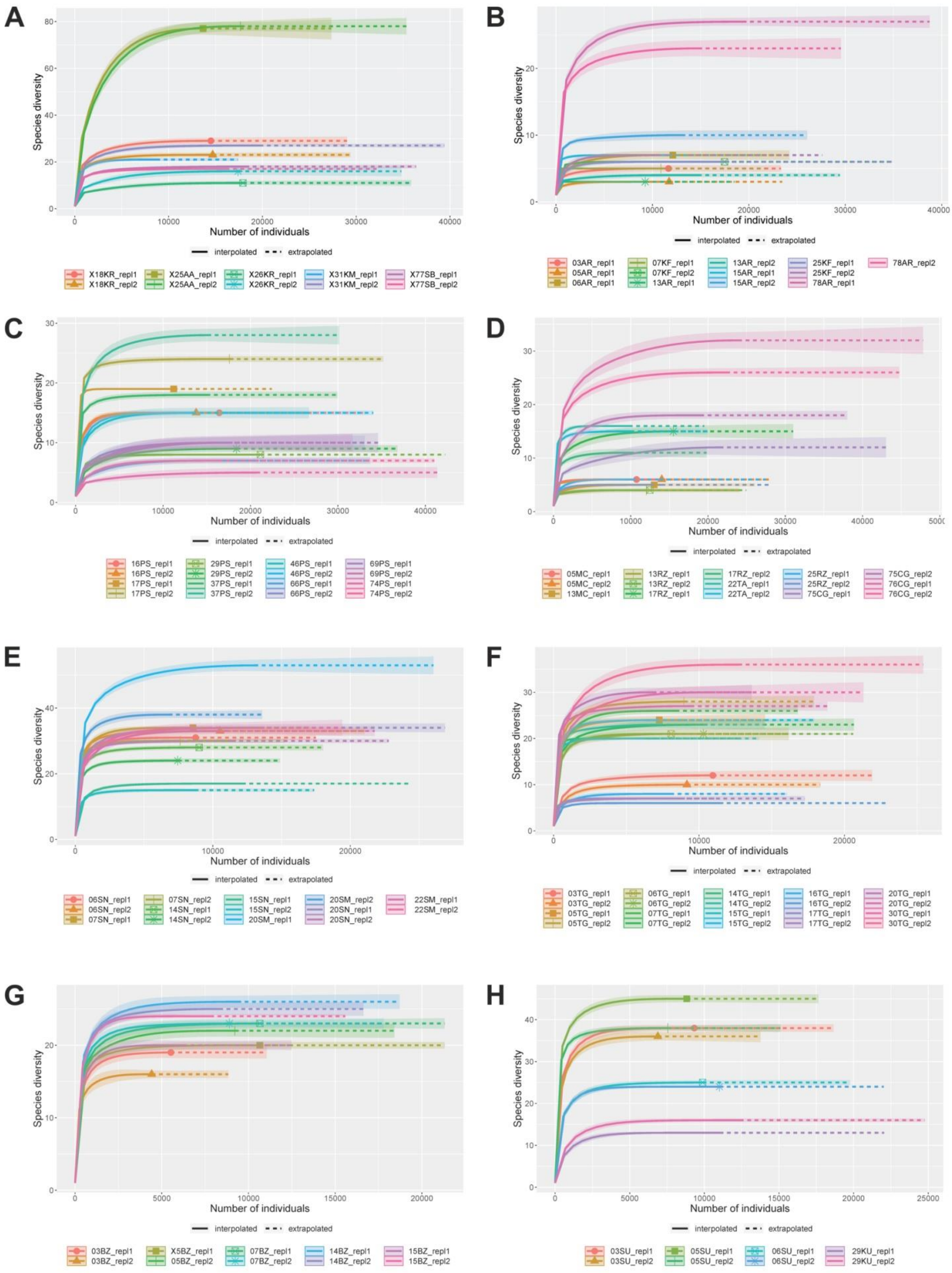

Supplementary Figure S1. Rarefaction curves calculated for 55 fermented milk product samples. A - curves for aarts (AA), khurunga (KR), koumiss (KM) and shubat (SB) samples; B – curves for ayran (AR) and kefir (KF) samples; C – curves for prostokvasha (PS) samples; D – curves for matsoni-like product (MC), ryazhenka (RZ), tan (TA) and chegen (CG) samples; E – curves for sour-cream (SN+SM) samples; F – curves for cottage cheese (TG) samples; G – curves for bryndza (BZ) samples; H – curves for suluguni-like cheese (SU) and khuruud (KU) samples.

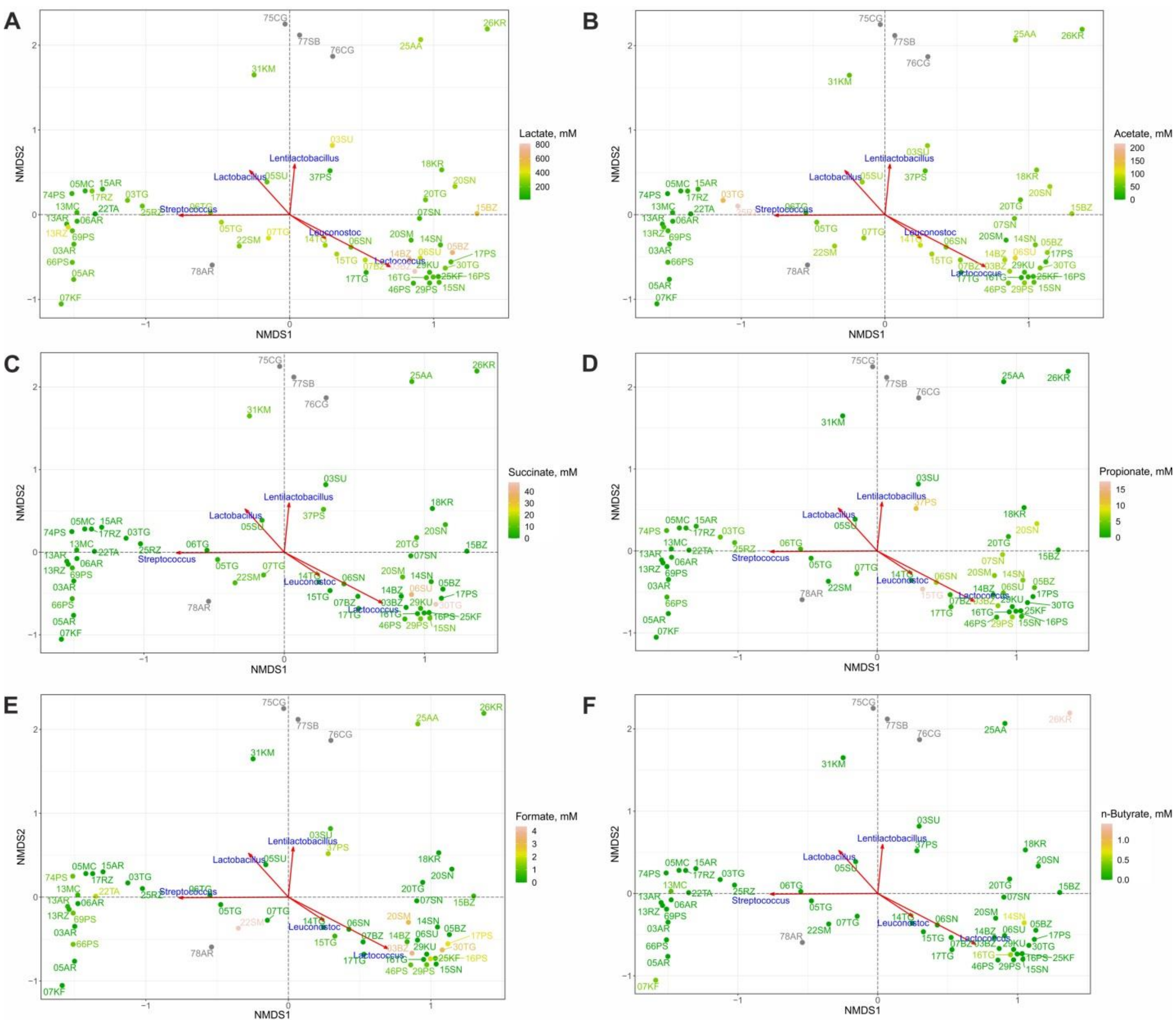

Supplementary Figure S2: FMP microbiomes dissimilarity displayed by NMDS plots (stress value = 0.13). Color grading corresponds to the concentrations of lactate (A), acetate (B), succinate (C), propionate (D), formate (E), *n*-butyrate (F). Gray color of samples indicates that VFA concentration was not measured in these samples. Red vectors indicate the correlation of the dominant genera abundance with the axes of ordination and their statistical significance based on a permutation test (1000 permutations, P-value < 0.05) i. e., demonstrate the strength and direction (within the present coordinates) of influence of certain bacterial taxa on samples clustering.

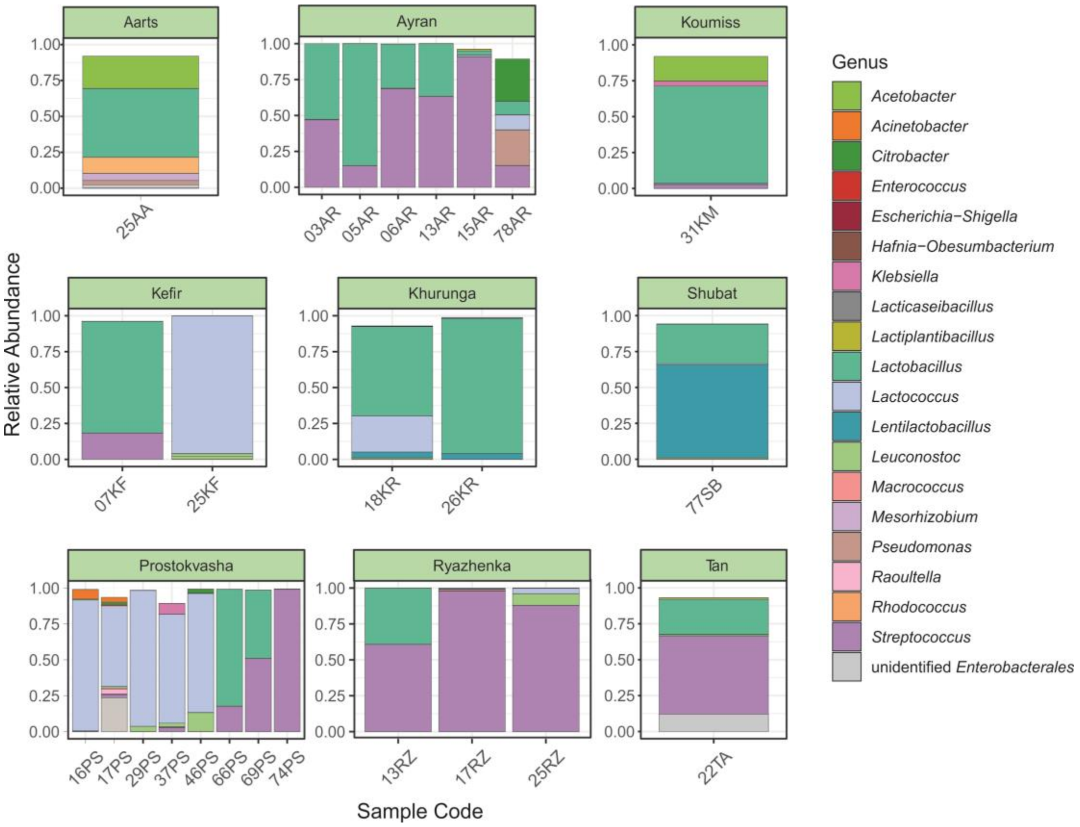

Supplementary Figure S3. Distribution of the 62 most abundant ASV on the genus level for beverages.

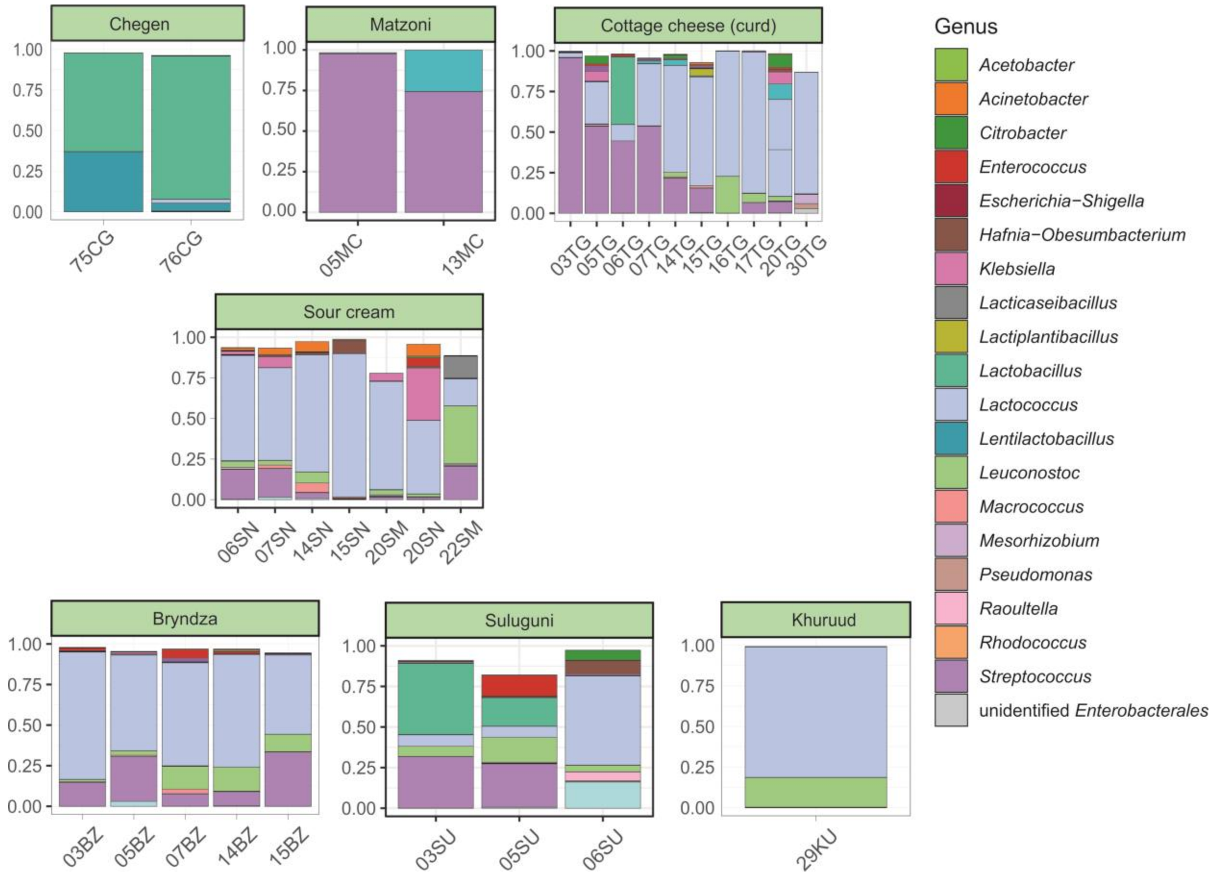

Supplementary Figure S4. Distribution of the 62 most abundant ASV on the genus level for cheeses and creamy products.
